# The Use of Small Electronic Devices and Health: Feasibility of Interventions for a Forthcoming Crossover Design

**DOI:** 10.2196/20410

**Published:** 2021-01-04

**Authors:** Lisbeth Hoekjaer Larsen, Maja Hedegaard Lauritzen, Sirin Wilhelmsen Gangstad, Troels Wesenberg Kjaer

**Affiliations:** 1 Department of Neurology Zealand University Hospital Roskilde Denmark; 2 Department of Applied Mathematics and Computer Science Technical University of Denmark Lyngby Denmark; 3 Uneeg Medical Lynge Denmark; 4 Faculty of Health and Medical Sciences University of Copenhagen Copenhagen Denmark

**Keywords:** accelerometer, activity trackers, aerobic capacity, insufficient sleep, media use, screen time, sleep problems, smartphones, wearable tracking devices

## Abstract

**Background:**

Modern lifestyle is heavily affected by technology such as smartphones, tablets, and other small computers; yet it remains unclear how our health and well-being are affected by the heavy use of these devices.

**Objective:**

This feasibility study aims to test two different interventions of an experimental protocol for a forthcoming large-scale community-based study and get estimates of parameters for sample size calculation. The aim of the large-scale study is to investigate the effect of (1) a wearable tracking device on aerobic capacity (VO_2_max/kg) and the effect of (2) restricting media use on total sleep time.

**Methods:**

Twenty healthy participants were included and equipped with a wrist-worn device tracking physical activity and sleep. Participants were allocated to either a physical activity group, which was instructed to use the wrist-worn device to support exercise, or a sleep silent group, which was instructed to remove or switch off all electronic devices in the bedroom (except the wrist-worn tracking device). The intervention lasted approximately 4 weeks. Data collected included blood pressure, submaximal cycle ergometer test, self-reported technology use, and compliance of using the wearable tracking device.

**Results:**

All participants wore the wearable tracking device 95.8% (SD 4.4%) of the time. Participants in the physical activity group increased aerobic capacity from 30.38 (SD 8.98) to 32.1 (SD 8.71) mL/kg/min (*t*=–2.31, *P*=.046) and decreased their systolic blood pressure from 126.5 (SD 15.8) mm Hg to 121.8 (SD 11.7) mm Hg (*t*=2.72, *P*=.02). The sleep silent group prolonged their time offline before bedtime from 18.1 (SD 19.4) minutes to 27.2 (SD 17.3) minutes (*t*=–2.94, *P*=.02).

**Conclusions:**

The two interventions are feasible to conduct. Participants were willing to wear the tracking device on their wrist and restrict all media use in their bedroom and thereby reduce bedtime technology use. Our results also suggest that tracking physical activity using a wearable device is accompanied by noteworthy health benefits. We outline necessary adjustments for a forthcoming large-scale study.

## Introduction

Progress in technology has revolutionized the way we live in modern society. Small and convenient electronic devices are with us everywhere and play a central role in our lives and the way we work, communicate, interact, search for information, do chores, and pass time. Yet it remains unclear how our health and well-being are affected by the use of these devices. In this feasibility study, we test an experimental protocol designed to investigate how the use of a wearable tracking device (WTD) and bedtime technology use affect physical activity and sleep, respectively. More knowledge of the effect of technology is needed as inactivity and insufficient sleep pose serious public health implications in modern society.

In western culture, physical inactivity and sedentary lifestyle are increasing and, as a consequence, so are health-related problems and health care costs [1]. Global Health Observatory data estimates that 37% of the adult population in high-income countries is insufficiently physically active [2]. It has been suggested that WTDs may encourage physically active behavior [3]. WTDs are wearable computers able to monitor different health-related parameters such as steps, distance covered, and pulse continuously under real-life conditions, and they are already widely used by consumers. The self-monitoring is made possible by different sensors and algorithms and is often accompanied by mobile apps. Modern WTDs have the opportunity to incorporate principles for behavior change in the promotion of physical activity including feedback, tailored information, gamification, rewards, goal setting, prompts, social comparison, and connectivity [3,4]. Despite the promising features embedded in WTDs, results are mixed from previous studies investigating the effect of increasing physical activity with WTDs on different health parameters [5-7]. Part of the discrepancy between studies may relate to study populations, interventions, comparators, and outcomes. The effect of using WTD on VO_2_max seems to be less studied, although this health parameter is known to be an important indicator of health-risk status. Epidemiologic studies have reported that a low VO_2_max is a more powerful predictor of risk for adverse outcomes than traditional risk factors, including hypertension, lipid abnormalities, smoking, physical inactivity, obesity, and diabetes mellitus [1,8,9].

Insufficient sleep constitutes another health risk in modern society. Recent evidence demonstrates the proportion of people getting less than the recommended hours of sleep is rising [10]. A survey conducted by the National Sleep Foundation found that the proportions of people sleeping fewer than 7 hours are 40% in Japan, 27% in the United States, and 21% in Germany [11]. Insufficient sleep can have multiple negative consequences, such as cognitive impairment, obesity, hypertension and insulin resistance (diabetes), and substantial economic losses [10,12]. It has been proposed that the increased use of media via smartphones, tablets, and other handheld devices before bedtime is worsening the challenge because the screen light significantly suppresses the secretion of melatonin and consequently disrupts sleep [13]. Furthermore, the contents received from these handheld devices may induce arousal and stress reactions, making it difficult to fall asleep [14]. Studies on smartphone use and sleep have quite consistently shown an association between bedtime technology use and sleep descriptors [14-16]. According to a study from Denmark, 40% of 815 young Danish students gave likes or sent messages during the night [17]. It is, however, unclear from this study and many similar studies whether smartphone activity is causing an increase in sleep onset latency and sleep interruption or if smartphone activity is used as an entertainment device among those with sleep impairment due to other causes [14,17]. Of note, a study with 942 Canadian students demonstrated that sleep problems predicted media use and not the opposite [18]. Most studies today are based on cross-sectional design, meaning that the causality is difficult to ascertain [14]. Recruiting participants for an experimental protocol may pose a challenge due to a lack of motivation to negotiate changes in bedtime smartphone use [19]. Nevertheless, more experimental research is needed on how bedtime uses of smartphones affect sleep measured over a longer period of time.

The purpose of this study was to test the feasibility of two different interventions of an experimental protocol and to get estimates of parameters for sample size calculation in order to refine the protocol for a forthcoming large-scale study. The aim of the forthcoming study is to investigate the effect of (1) using a WTD on aerobic capacity and (2) removing electronic devices from the bedroom on total sleep time (TST). The forthcoming study will contain both a baseline and an intervention period, but this study aims at investigating the feasibility of the interventions only.

## Methods

### Participants

Twenty able-bodied participants (4 males, with a mean age of 48 [SD 9] years) were recruited to participate in the study through local advertisement in the municipality of Naestved, Denmark. Participants were required to be aged 18 to 75 years, to own a smartphone or tablet, and to be able to exercise on their own. People already exercising for more than 15 hours weekly were not eligible. The sample size for this study was set to 20 participants, which we estimated to be adequate to test the experimental protocol and get estimates of parameters for sample size calculation to the necessary degree of precision [20]. All participants gave informed consent to the experimental procedure, which was approved by the local ethics committee (SJ-743). The study was performed in accordance with the Declaration of Helsinki.

### Experimental Protocol

**Figure 1:**
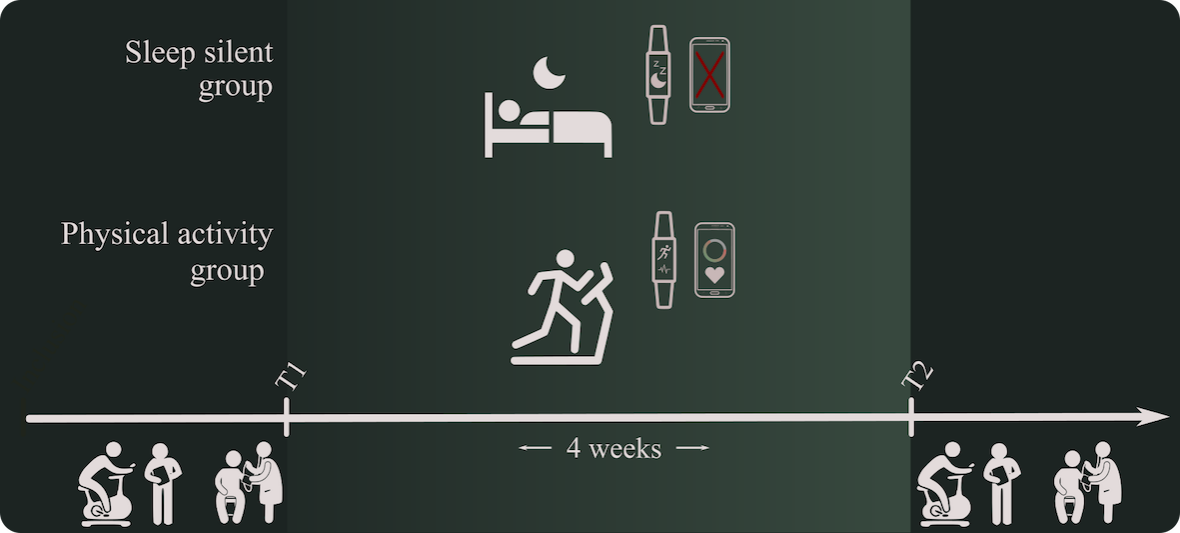
Experimental setup.

Participants attended 2 test days (T1 and T2) with 33 (SD 8) days in between (Figure 1). BMI, blood pressure (BP, mean of 3 repeated measures), and a submaximal cycle ergometer test to estimate VO_2_max [21] were conducted at both test days. Furthermore, participants answered questions on a tablet regarding their level of moderate-to-vigorous physical activity (MVPA) in minutes per week with the Nordic Physical Activity Questionnaire-short (NPAQ-short) [22], their current level of sleep problems with the Insomnia Severity Index (ISI) [23], and their time offline (TO) before and after sleep. The NPAQ-short is a 2-item questionnaire to monitor physical activity (time and intensity) and compliance with the World Health Organization (WHO) recommendations. The ISI is a 7-item questionnaire where participants rate symptoms of their sleep problems using a Likert-type scale. Each item is rated on a 0 to 4 scale, and the total score ranges from 0 to 28. A higher score suggests more severe insomnia.

On T1, participants were allocated to either a physical activity (PA) group or a sleep silent (SS) group by minimization in order to ensure a balanced age distribution in both groups. This was done to examine the feasibility of both interventions among different age groups. In the PA group, participants were encouraged to challenge themselves with a realistic self-chosen fitness goal for the intervention period based on their resources and prior training level. The goal could be specific, such as accomplishing running 5 kilometers without stopping, or more general, such as meeting WHO’s minimum recommendation of 150 minutes MVPA weekly [24]. Participants in the PA group were introduced to the WTD and instructed to follow their progress on the accompanying mobile app Garmin Connect. All exercise was performed independently by the participants. Participants were asked after the intervention about their use of WTD and whether they wished to continue using a wrist-worn tracking device in the future. The SS group was instructed to remove or switch off all electronic devices in the bedroom (except the WTD). Several different technologies (such as computers, tablets, and other handheld devices) are used for the same activities as a smartphone and, therefore, use of all electronic devices was restricted. Analog alarm clocks were distributed, and participants were asked not to check their smartphone and other digital screens and devices from bedtime until they get up in the morning.

On T1, all participants were equipped with a WTD (Vivosmart 4, Garmin Ltd), and instructed to download the mobile app Garmin Connect and set up a user account. One participant already used a WTD (Fenix 5X, Garmin Ltd), which the participant continued to use instead of Vivosmart 4. All participants were instructed to wear their WTD on their wrist for the entire period of approximately 4 weeks. The small device detects physical activity, heart rate, and sleep via an embedded triaxial accelerometer, optical photoplethysmography signals, and associated algorithms. It automatically records intensity and type and duration of different activity patterns such as walking, running, and biking for at least 10 minutes and attempts to detect sleep onset, sleep end, sleep stages (light, deep, rapid eye movement, and wake), and level of movement during sleep. Based on the time stamps of the WTD measurements, the compliance of wearing the WTD was investigated for each participant. The amount of time a pulse measurement was available in the recorded data relative to the length of the intervention period was computed. The pulse was chosen as it is sampled relatively frequently (1 sample per 2 minutes). The percentage of available pulse data was used as a proxy for the percentage of time the participant wore the WTD. Furthermore, the amount of nights with missing TST estimates in the WTD recordings was investigated, as the TST is an important parameter in the upcoming study.

## Results

**Figure 2:**
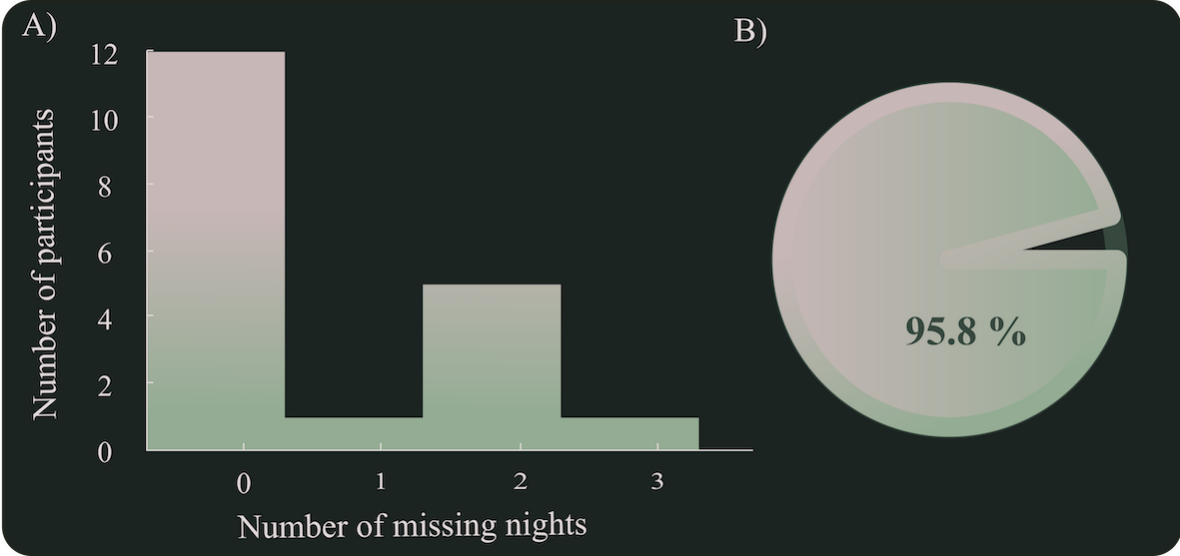
Compliance with wearing the wearable tracking device. A) shows number of missing nights per participant, and B) shows compliance in percentages for all participants during the entire intervention period.

Twenty participants were recruited for this study. One participant from the SS group lost the WTD after 3 weeks and was excluded. Another participant from the SS group got an injured little finger while walking a dog (not related to study activities) and therefore did not perform the cycle ergometer test at T2. Throughout the intervention period, the participants wore the WTD device 95.8% (SD 4.4%) of the time. Seven participants missed 1 to 3 nights of data due to not charging the battery (Figure 2).

From T1 to T2, the PA group increased their estimated VO_2_max from 30.38 (SD 8.98) to 32.1 (SD 8.71) mL/kg/min (*t*=–2.31, *P*=.046) and the systolic BP decreased from 126.5 (SD 15.8) mm Hg at T1 to 121.8 (SD 11.7) mm Hg at T2 (*t*=2.72, *P*=.02) while no difference was observed in the diastolic BP (from 84.3 [SD 10.1] mm Hg to 80.4 [SD 8.8] mm Hg; *t*=1.96, *P*=.08) or in the BMI (see Table 1). According to self-reported exercise behavior, 2 participants in the PA group did not meet the WHO’s minimum recommendation of 150 MVPA minutes per week at T1, while 2 participants had filled out the questionnaire incorrectly. At T2, 1 participant in the PA group reported an activity level below 150 MVPA minutes per week and no difference was reported in MVPA between T1 and T2 in the PA group (from 310 [SD 216] to 375 [SD 172] minutes per week, *t*=–1.5, *P*=.18; Table 1). Three participants in the PA group reported sleep problems (ISI value 8.3 [SD 2], n=3) at T1 and 2 participants (ISI value 4.7 [SD 4], n=3) at T2. No change was observed in time offline (TO) in the PA group (Table 1). All participants in the PA group reported to use the WTD to track their activity level in the intervention period. Eight participants wished to continue using a WTD after T2, while 2 participants were reluctant due to a lack of interest in the information collected and stress associated with self-monitoring respectively.

In the SS group, 6 participants reported sleep problems (ISI value 9 [SD 2], n=6) at T1 and 3 participants (ISI value 4.7 [SD 6], n=6) at T2. The SS group prolonged the TO before bedtime (from 18.1 [SD 19.4] to 27.2 [SD 17.3] minutes; *t*=–2.94, *P*=.02) while no change was observed in TO in the morning (Table 2). In the SS group, no change was observed in the estimated VO_2_max, BP, or BMI between test days (Table 2). According to self-reported exercise behavior, 3 participants in SS group did not meet the WHO’s minimum recommendation of 150 MVPA minutes per week at both T1 and T2, and no change in the MVPA was observed in the group (from 274 [SD 172] to 283 [SD 203] minutes per week; *t*=–0.18, *P*=.86). One participant had filled out the questionnaire incorrectly.

**Table 1:** Pre-post measurements in the physical activity group.

Characteristic	T1, mean (SD)	T2, mean (SD)	*t*	*P* value
BMI	26.73 (4.79)	26.69 (4.77)	0.61	.56
Systolic BP^a^ (mm Hg)	126.5 (15.77)	121.8 (11.71)	2.72	.02
Diastolic BP (mm Hg)	84.3 (10.05)	80.37 (8.75)	1.96	.08
VO_2_max^b^/kg	30.38 (8.98)	32.1 (8.71)	–2.31	.046
SR MVPA^c^ (min/week)	310 (216)	375 (172)	–1.50	.18
SR TO^d^, evening (min)	11.4 (15.55)	15.1 (16.67)	–1.13	.29
SR TO, morning (min)	21.9 (28.5)	22.8 (29.78)	–0.14	.89

^a^BP: blood pressure.

^b^VO_2_max: maximal oxygen uptake.

^c^SR MVPA: self-reported moderate-to-vigorous physical activity.

^d^SR TO: self-reported time offline.

**Table 2:** Pre-post measurements in the sleep silent group.

Characteristic	T1, mean (SD)	T2, mean (SD)	*t*	*P* value
BMI	27.35 (5.59)	27.30 (5.6)	0.26	.80
Systolic BP^a^ (mm Hg)	127.15 (16.9)	125.78 (17.78)	0.30	.77
Diastolic BP (mm Hg)	84.67 (10.41)	87.41 (11.58)	–1.16	.28
VO_2_max^b^/kg	30.42 (8.57)	32.18 (9.25)	–1.31	.23
SR MVPA^c^ (min/week)	274 (172)	283 (203)	–0.18	.86
SR TO^d^, evening (min)	18.1 (19.36)	27.22 (17.34)	–2.94	.02
SR TO, morning (min)	28 (28.08)	32.22 (19.70)	–1.81	.11

^a^BP: blood pressure.

^b^VO_2_max: maximal oxygen uptake.

^c^SR MVPA: self-reported moderate-to-vigorous physical activity.

^d^SR TO: self-reported time offline.

## Discussion

### Principal Findings

Results from this study suggest that the experimental protocol is feasible to conduct: participants were willing to wear the wrist-worn tracking device and keep track of their exercise or remove their smartphone from the bedroom. The participants wore the WTD nearly 96% of the time they were enrolled in the study, demonstrating an extremely high compliance considering the participants wear the WTD around-the-clock and it includes an inevitable loss of data points due to necessary charging of battery one or twice a week.

The majority (60%) of participants in this feasibility study had a low or somewhat low VO_2_max at T1 in both groups according to Astrands classification of aerobic capacity by age and gender [25]. It has been demonstrated that a low VO_2_max is associated with a 2- to 5-fold increase in cardiovascular disease or all-cause mortality, independent of other cardiovascular disease risk factors [26]. Importantly, relatively small improvements in aerobic capacity such as 1 metabolic equivalent (3.5 mL/kg/minute) have been associated with 8% to 35% reductions in mortality [26]. From this perspective, an average VO_2_max increase of 1.71 mL/kg/minute in the PA group could suggest a noteworthy health benefit if the participants maintain the level of exercise from the intervention period in future.

In the PA group we also observed an average decrease of systolic BP of 4.7 mm Hg. Hypertension significantly increases the risks of heart, brain, and other diseases. In a meta-analysis by Lewington et al [27], the age-specific relevance of usual BP to vascular mortality was assessed from one million adults in 61 prospective studies. The authors found that a reduction in systolic BP of just 2 mm Hg reduces apoplexy mortality by 10% and death of ischemic heart disease by 7% among middle-aged people. In light of this, our observed decrease in average systolic BP of 4.7 mm Hg is also highly relevant. A recent review evaluated the effect of using WTDs on metabolic outcomes such as BP, blood glucose level, and cholesterol levels in patients [5]. Based on the 6 included studies, the authors conclude that WTDs play a role as a facilitator in motivating and accelerating physical activity, but current data do not suggest other consistent health benefits for patients. Two other recent reviews conclude that people using wearable devices improved their daily step counts regardless of age, sex and health status [6,7]. Of note, Brickwood et al [7] also found a significant increase in MVPA, while Lynch et al [6] did not find this positive effect. A great challenge in this field is that the literature remains limited primarily to short-term studies, and many of these are underpowered feasibility or pilot studies [5,28]. Personal preferences and adverse effects related to self-monitoring may also play a role in the disagreement between studies. For instance, modern WTDs allow individuals to gain insight into their own activity level 24 hours a day, and studies have demonstrated that for some individuals self-monitoring is valued and can prompt further goal-directed behavior while for other individuals the inability to meet goals can trigger negative experiences [3]. Two participants in the PA group reported skepticism to continue using a WTD due to a lack of interest in measurements and stress associated with self-monitoring, respectively. Thus, the effect of self-monitoring of PA behavior may be affected by personality. Large studies that can accommodate the fast pace of advances in technology are needed to examine if WTDs can enhance important health outcomes and determine which populations are most receptive to WTDs.

Participants in the SS group prolonged the TO before bedtime, demonstrating a willingness to incorporate restrictions on bedtime technology use. Previous studies suggest that bedtime technology use is negatively related to sleep outcomes, but few longitudinal studies have been conducted with an experimental setup. A strength of this study is therefore the interventional and feasible study design. The few existing experimental studies that have been conducted report contradictory results on sleep measures [19,29-31]. For instance, restricting mobile phone use before bedtime for 4 weeks had no effect on sleep measures in a study conducted by Harris el al [29] in Norwegian high school athletes, while He et al [30] found several improvements in both sleep measures and working memory in Japanese university students. Of note, the inclusion criteria differed in the 2 studies: He et al [30] only included participants with poor sleep and a habit of using a mobile phone during bedtime, while Harris et al [29] did not have such inclusion criteria. In our feasibility study, we did not have any inclusion criteria regarding smartphone use meaning that we included both light and heavy smartphone users. A recent telephone-based survey showed that 42% of participants reported using electronic devices in bed after lights out, and 27% of adults who reported always using electronic devices in bed were spending over an hour per night using them [32]. The survey demonstrates a large variance in the habits of bedtime technology among adults, which is important to consider in order to illuminate how the use of smartphones affect sleep and sleep quality.

Current research on the associations between sleep measures and smartphone use has mainly focused on children, adolescents, or university students, which compromises generalizability of the results to the population above 25 to 30 years [14,32]. In our feasibility study, the average age of participants was 48 years ranging from age 24 to 60 years. This may also explain why we did not encounter similar challenges in the recruiting process as Bartel et al [19], who only included adolescents. Nevertheless, a study including all age groups can contribute to cover a gap in the literature.

The effect of bedtime technology use has mainly been investigated with self-reported outcome measures and may thereby be prone to misclassification, recall difficulty, recall bias, and response-style bias [14]. Only a few studies have applied objective sleep measures based on actigraphy and examined the association between sleep and self-reported media use [33,34]. These studies report that self-reported bedtime technology use is negatively related to objective sleep measures in adolescents. Although the literature shows that actigraphy reliably detects sleep-wake patterns in normal individuals [35], we are currently investigating the validity of the sleep detection provided by the Vivosmart 4 in a separate study. One study has used a screen time detecting app to examine the relationship between self-reported sleep and screen time measured objectively. Increased screen time was associated with poor self-reported sleep outcomes (sleep quality, sleep duration, sleep efficiency, and longer sleep onset latency) [36]. Future research should ideally combine a large-scale intervention with objective measures of both sleep and screen time in an adult population in order to draw valid conclusions about cause and effect of the association between bedtime technology use and sleep measures.

### Limitations

The feasibility study design had some limitations, which preferably should be adjusted in the forthcoming large-scale study. First, we did not have any inclusion criteria regarding usual smartphone use and physical activity level, meaning that for some participants the intervention made little change to their established pattern. An advantage of such broad inclusion criteria is that it enables a generalization to a broad population group. However, a disadvantage is that the result may be contaminated. Hence, in the large-scale study inclusion criteria should be added in order to ensure examination of relevant participants and the content of the interventions needs to be specified. Second, this study design did not include actual control observations, which is necessary in order to determine an effect of an intervention. Finally, the intervention period of 4 weeks is short and should be expanded in order to investigate long-term effects. Meeting these limitations in a forthcoming large-scale study can contribute with experimental evidence of the effect of using WTDs on aerobic capacity and restricting bedtime technology use on sleep length.

### Conclusions

The experimental protocol in this study was feasible to conduct. Participants were willing to wear the WTD around-the-clock and use the wrist-worn device to support exercise or remove their smartphone from the bedroom. We observed that tracking PA using a wearable device is accompanied by noteworthy health benefits and that restricting technology use in the bedroom reduce participant use of bedtime technology. In a forthcoming large-scale study, sample size calculations will be based on collected estimates of VO_2_max and TST. Furthermore, in order to obtain experimental evidence of the effect of using WTDs on aerobic capacity and illuminate causal claims of restricting bedtime technology use on TST, adjustment highlighted in the previous section should be prioritized.

## Abbreviations

BP: blood pressure
ISI: Insomnia Severity Index
MVPA: moderate-to-vigorous physical activity
NPAQ-short: Nordic Physical Activity Questionnaire-short
PA: physical activity
SS: sleep silent
T1: day 1
T2: day 2
TO: time offline
TST: total sleep time
VO_2_max: maximal oxygen uptake
WHO: World Health Organization
WTD: wearable tracking device
